# Structural insights into MltB from *Acinetobacter baumannii*: a conserved catalytic tunnel with unique domain arrangement

**DOI:** 10.1107/S2052252526002770

**Published:** 2026-04-09

**Authors:** Hyunseok Jang, Chang Min Kim, Chang Sup Lee, Hyun Ho Park

**Affiliations:** a College of Pharmacy Chung-Ang University Seoul 06974 Republic of Korea; b Department of Global Innovative Drugs Graduate School of Chung-Ang University Seoul 06974 Republic of Korea; c Department of Molecular Genetics University of Texas Southwestern Medical Center Dallas TX 75390 USA; d College of Pharmacy and Research Institute of Pharmaceutical Sciences Gyeongsang National University Jinju52828 Republic of Korea; Chinese Academy of Sciences, China

**Keywords:** *Acinetobacter baumannii*, MltB, lytic transglycosylases, antimicrobials

## Abstract

Lytic transglycosylases (LTs) play a central role in bacterial cell wall remodeling by cleaving the β-1,4-glycosidic bond in peptidoglycan via a non-hydrolytic mechanism, producing 1,6-anhydroMurNAc termini crucial for recycling and signaling. Among LTs, membrane-bound lytic transglycosylase B (MltB) contributes to peptidoglycan turnover, antibiotic resistance and protein–protein interactions. Despite its importance, the structural basis of MltB function in *Acinetobacter baumannii*, a multidrug-resistant pathogen, remains unclear. Here, we report the crystal structure of abMltB at 1.79 Å resolution. abMltB adopts a monomeric structure comprising three domains: an N-terminal domain (NTD), a catalytic domain (CD) and a C-terminal domain (CTD). The NTD connects to the CD via a flexible loop that partially occludes the active site, forming a tunnel-like cavity. Comparative analysis with homologous enzymes such as SltB1 and Slt35 reveals conserved catalytic residues, including Glu129 and key aromatic residues, while the CTD in abMltB adopts a distinct orientation. Notably, although calcium ions stabilize related enzymes, no metal binding was observed in abMltB. These findings highlight conserved mechanistic features and unique structural adaptations of abMltB, offering insight into its functional role in *A. baumannii* and potential as a novel antimicrobial target.

## Abstract 2

We characterized MltB from the human pathogen *Acinetobacter baumannii* and unveiled its high-resolution crystal structure, revealing that abMltB features three domains forming a tunnel-like catalytic cavity. Structural comparison with related enzymes reveals conserved catalytic residues and a unique C-terminal domain orientation. Based on the current structural and biochemical studies, we modeled the working mechanism of abMltB.

## 1. Introduction

**Figure 1:**
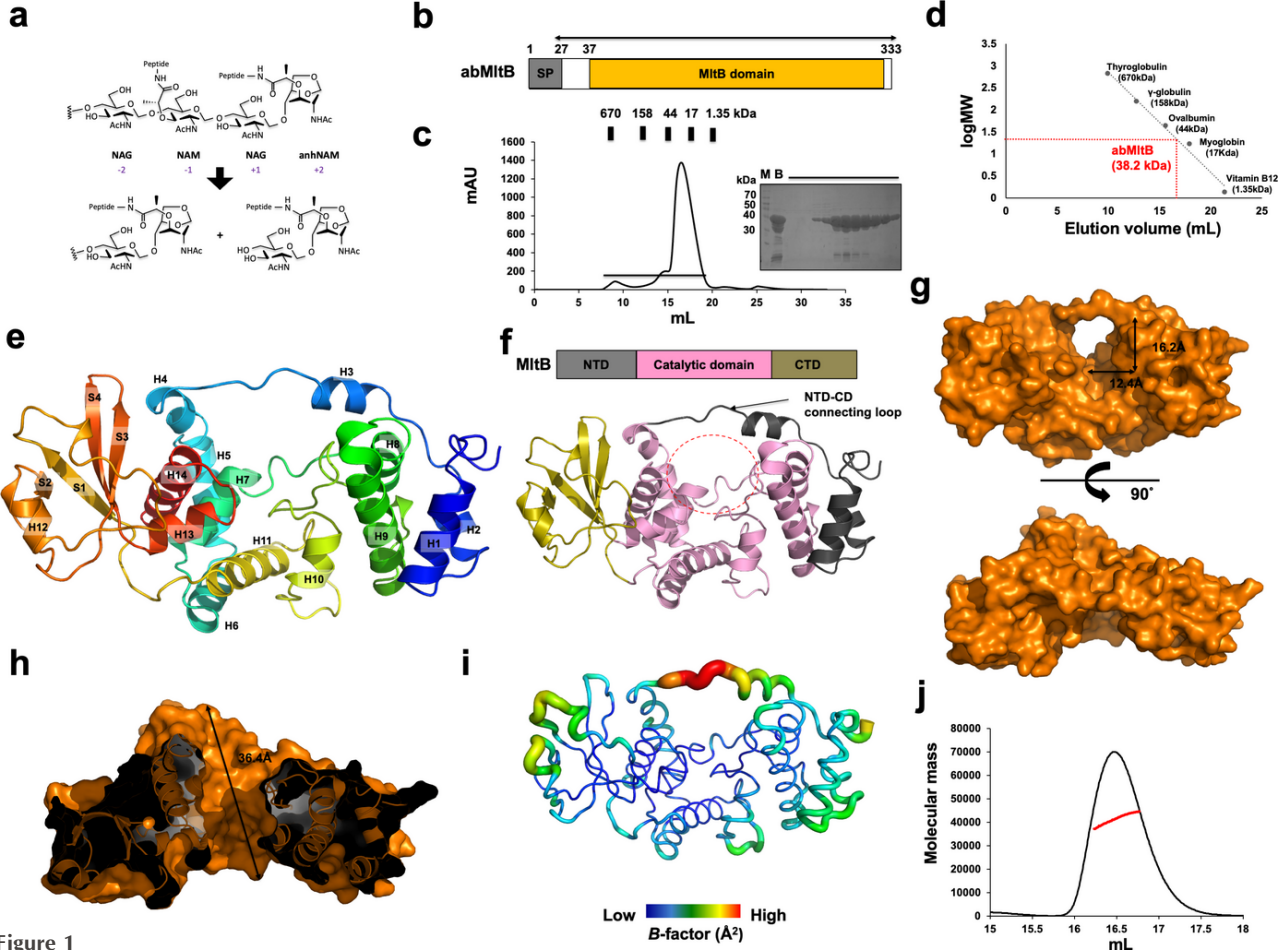
Crystal structure of abMltB. (*a*) Overall reaction of lytic transglycosylases. (*b*) Schematic representation of the overall domain architecture of MltB. (*c*) Size-exclusion chromatography (SEC) profile of abMltB. SDS–PAGE results used to verify the identity and purity of the protein are displayed adjacent to the major peak, with the loaded fractions highlighted by a black line. M and B indicate marker and before-loaded sample, respectively. (*d*) The elution volume line fitting in SEC plotted against the size marker and the logarithm of the molecular weight of abMltB. The red point on the fitting line signifies the elution volume of abMltB. The molecular weights of the size markers are indicated along the standard line for reference. (*e*) Rainbow-colored cartoon model of monomeric abMltB, with a color gradient from blue (N-terminus) to red (C-terminus). Helices and strands are labeled ‘H’ and ‘S’, respectively. (*f*) Domain organization within the abMltB structure, highlighting the boundary between domains. A putative substrate-binding pocket is marked by a red dotted circle. (*g*) Surface model of abMltB. (*h*) Cross-sectional view of the abMltB structure. (*i*) *B*-factor distribution visualized using a putty model, with colors ranging from blue (low *B* factor) to red (high *B* factor). (*j*) Multi-angle light scattering (MALS) profile derived from the main size-exclusion chromatography (SEC) peak. The red line indicates the experimental molecular mass analyzed by MALS.

Bacterial cell wall remodeling is a dynamic and tightly regulated process essential for maintaining cell shape, integrity and survival under various environmental conditions (Mueller & Levin, 2020 ▸). One of the key players in this process is the family of lytic transglycosylases (LTs), a group of enzymes that cleave the β-1,4-glycosidic bond between *N*-acetylmuramic acid (MurNAc) and *N*-acetylglucosamine (GlcNAc) residues in the peptidoglycan backbone [Fig. 1 ▸(*a*)] (Blackburn & Clarke, 2001 ▸; Lee *et al.*, 2013 ▸; Lommatzsch *et al.*, 1997 ▸). Unlike lysozymes, LTs catalyze this cleavage via a non-hydrolytic, intramolecular transglycosylation mechanism, resulting in the formation of 1,6-anhydroMurNAc termini which are key intermediates in peptidoglycan recycling and signaling (Blackburn & Clarke, 2001 ▸; Lee *et al.*, 2013 ▸; Lommatzsch *et al.*, 1997 ▸).

Among the various LTs, membrane-bound lytic transglycosylase B (MltB) has attracted increasing attention due to its role in cell wall turnover, cell division, antibiotic resistance, and interaction with β-lactamases and other peptidoglycan-binding proteins (Martinez-Bond *et al.*, 2022 ▸; Crépin *et al.*, 2018 ▸). In Gram-negative pathogens such as *Escherichia coli* and *Pseudomonas aeruginosa*, MltB is known to participate in controlled degradation of the cell wall during growth and in response to antibiotic stress (Suvorov *et al.*, 2008 ▸; Dhar *et al.*, 2018 ▸). However, in *Acinetobacter baumannii*, an opportunistic pathogen notorious for its multidrug resistance and persistence in clinical settings, the structure and mechanistic function of MltB remain poorly understood.

Previous structural studies of other LTs have provided valuable insights into the conserved catalytic architecture and substrate recognition mechanisms of this enzyme family (van Asselt *et al.*, 1999 ▸; Lee *et al.*, 2024 ▸; Domínguez-Gil *et al.*, 2017 ▸; Jang *et al.*, 2021 ▸; Lee *et al.*, 2017 ▸). These enzymes typically share a conserved SLT (soluble lytic transglycosylase) domain fold, but they differ in their modular organization, periplasmic localization, substrate specificity and regulatory roles (Alcorlo *et al.*, 2017 ▸; Blackburn & Clarke, 2001 ▸; Dik *et al.*, 2017 ▸). For instance, SltB1 from *E. coli* and Slt35 from *P. aeruginosa* have been shown to exhibit subtle variations in their active site residues and overall domain orientation, which may reflect differences in their physiological roles or substrate preferences (Domínguez-Gil *et al.*, 2017 ▸; van Asselt *et al.*, 1999 ▸).

Despite these advances, a comprehensive structural comparison of MltB from *A. baumannii* with these functionally related enzymes has not yet been reported. Understanding these structural similarities and differences is critical, not only for elucidating the molecular basis of MltB function in *A. baumannii*, but also for identifying novel targets for the development of antimicrobial agents aimed at weakening the bacterial cell envelope. In this study, we present the first high-resolution tertiary structure of MltB from *A. baumannii* (hereafter called abMltB), and perform a comparative structural analysis with previously solved homologs including Slt35, SltB1 and SltB3. The crystal structure of abMltB showed that it is composed of three distinct domains: an N-terminal domain (NTD), a catalytic domain (CD) and a C-terminal domain (CTD). The NTD is connected to the CD via a long, flexible loop that overlays the putative active site, forming a deep tunnel-like cavity. Structural comparison with homologous lytic transglycosylases showed high overall similarity, though the CTD of abMltB is uniquely positioned farther from the catalytic domain. Sequence and structural alignments confirmed that key residues involved in substrate binding and catalysis – including the catalytic glutamate (Glu129) and several conserved aromatic residues – are preserved. Although Ca^2+^ binding is essential for stability in other homologs, no bound ion was observed in abMltB, suggesting that it remains stable without metal coordination. Collectively, these findings provide new structural insights into MltB function and suggest a conserved catalytic mechanism mediated by a tunnel-like cavity formed by the NTD–catalytic domain interface. Our findings reveal both conserved and unique features of the abMltB, offering new insights into its role in cell wall metabolism and its potential as a target for therapeutic intervention.

## 2. Materials and methods

### 2.1. Protein expression and purification

The expression construct encoding abMltB (residues Gln27–Arg333) was generated by subcloning a codon-optimized gene into the NdeI–XhoI sites of pET-28a. The resulting plasmid was transformed into *E. coli* BL21(DE3) by heat shock (42°C, 45 seconds), and transformants were selected on LB-agar plates containing 50 µg ml^−1^ kanamycin (Kan). A single colony was used to inoculate 10 ml LB media containing 50 µg ml^−1^ Kan and grown overnight at 37°C in the shaking (180 r.p.m.) incubator. The overnight culture was diluted 1:100 into 1 l LB containing 50 µg ml^−1^ Kan and incubated at 37°C until OD_600_ reached 0.6–0.8. Cultures were then chilled on ice for 15 min and induced with 1 m*M* IPTG, followed by overnight expression at 20°C (180 r.p.m.).

Cells were harvested by centrifugation (6000*g*, 10 min, 4°C), resuspended in 10 ml lysis buffer (20 m*M* Tris–HCl pH 8.0, 500 m*M* NaCl, 25 m*M* imidazole, 0.1 m*M* PMSF), and disrupted by sonication on ice (10 seconds on/20 seconds off cycles, total 5 min). The lysate was clarified by centrifugation (16 000*g*, 20 min, 4°C), and the supernatant was gently mixed with 2 ml Ni-NTA resin (Qiagen) for 2 h at 4°C. The resin was loaded into a gravity-flow column, washed with 30 ml washing buffer (20 m*M* Tris–HCl pH 8.0, 500 m*M* NaCl, 60 m*M* imidazole), and the bound target protein was eluted with 5 ml elution buffer (20 m*M* Tris–HCl pH 8.0, 500 m*M* NaCl, 250 m*M* imidazole).

For final polishing, the eluate was applied to a Superdex 200 Increase 10/300 GL column (GE Healthcare) on an ÄKTA explorer system, pre-equilibrated in size exclusion chromatography (SEC) buffer (20 m*M* Tris–HCl pH 8.0, 150 m*M* NaCl). Peak fractions corresponding to monomeric abMltB were pooled and concentrated to ∼10 mg ml^−1^ using a 30 kDa cutoff centrifugal concentrator.

### 2.2. Crystallization and data collection

The highest-quality crystals of abMltB were obtained at 20°C by sitting-drop vapor diffusion. In brief, 1 µl of protein solution (10.2 mg ml^−1^ in 20 m*M* Tris–HCl pH 8.0, 150 m*M* NaCl) was mixed with 1 µl of reservoir solution [25%(*w*/*v*) PEG 1500, 0.1 *M* MMT buffer pH 6.5] and equilibrated against a 100 µl reservoir. The crystals appeared after ∼30 days. Prior to data collection, crystals were briefly transferred into 10 µl of reservoir solution supplemented with 30%(*v*/*v*) glycerol as a cryoprotectant and flash-cooled in liquid nitrogen.

Diffraction data were recorded at 100 K on beamline 5C of the Pohang Accelerator Laboratory (PAL, South Korea) at a wavelength of 1.000 Å. Data reduction (indexing, integration, scaling) was carried out with *HKL-2000* (Otwinowski & Minor, 1997 ▸).

### 2.3. Determination and analysis of the structure

Initial phases were determined by molecular replacement in *MOLREP* (*CCP4*) using a homologous lytic transglycosylase (PDB ID: 5o8x) as the search model (Collaborative Computational Project, 1994 ▸). Automated model building was performed with *AutoBuild* (*Phenix*) (Adams *et al.*, 2010 ▸), followed by iterative cycles of manual adjustment in *Coot* (Emsley & Cowtan, 2004 ▸) and refinement with *REFMAC5* (Collaborative Computational Project, 1994 ▸) and *phenix.refine* (Adams *et al.*, 2010 ▸). The final model was validated with *MolProbity* (Chen *et al.*, 2010 ▸) and figures were prepared in *PyMOL* (DeLano & Lam, 2005 ▸).

### 2.4. SEC–multi-angle light scattering (MALS) analysis

For solution-state molar mass determination, purified abMltB was filtered (0.2 µm) and loaded onto a Superdex 200 Increase 10/300 GL column (GE Healthcare) equilibrated in SEC buffer (20 m*M* Tris–HCl pH 8.0, 150 m*M* NaCl). Multi-angle light scattering was measured with a DAWN TREOS detector (Wyatt Technology) in line with an ÄKTA explorer; bovine serum albumin served as the calibration standard. Absolute molecular weights were calculated using *ASTRA* (Wyatt Technology).

### 2.5. Sequence alignment

The amino-acid sequences of abMltB derived from various species were analyzed using *Clustal Omega* (https://www.ebi.ac.uk/Tools/msa/clustalo/).

### 2.6. Isothermal titration calorimetry (ITC)

A Nano ITC (TA Instruments) was used for the isothermal titration calorimetry experiments. Purified abMltB protein was dialyzed against PBS buffer with 0.5 m*M* EDTA, and Ca^2+^ was dissolved in the same buffer to minimize the heat of dilution values. Prior to titration, the protein samples and Ca^2+^ were centrifuged at 13 000 r.p.m. at 4°C for 10 min to remove all precipitants. For incremental injection into the Nano ITC, 2 µl of 1 m*M* Ca^2+^ was injected into a sample cell containing 180 µl of abMltB protein sample at a concentration of ∼20 µ*M*. The titration was carried out at 15°C with 18 injections at 160 s intervals. The area under each titration peak was integrated, plotted against the number of injections, and fitted with a one-site independent binding model, using the software provided by TA Instruments. Experimental data were subtracted from appropriate baselines acquired by injecting Ca^2+^ into the buffer, without the protein sample.

### 2.7. Accession codes

Coordinates and structure factors have been deposited in the RCSB Protein Data Bank with the PDB ID code 9vlr.

## 3. Results and discussion

### 3.1. Overall structure of abMltB

The limited amount of structural data on MltB largely stems from its poor solubility when membrane-anchored. To overcome this, we designed a codon-optimized construct lacking the native signal peptide (residues Gln27–Arg333), which yielded a highly soluble abMltB variant [Fig. 1 ▸(*b*)]. Using a streamlined two-step protocol – Ni^2+^ affinity chromatography followed by size-exclusion chromatography (SEC) – we obtained ∼10 mg of >90%-pure protein per litre of LB culture [Fig. 1 ▸(*b*), (*c*)]. SEC profiles revealed one distinct peak corresponding to monomeric abMltB [Fig. 1 ▸(*c*), (*d*)]. The 1.79 Å high-resolution crystal structure of abMltB was finally resolved and refined to *R*_work_ = 18.25% and *R*_free_ = 20.18%. The crystallographic and refinement statistics are summarized in Table 1 ▸.

One molecule was found in the asymmetric unit (ASU) [Fig. 1 ▸(*e*)]. The final model of the molecule was constructed from residue Gln27 to residue Arg333. The high-resolution crystal structure of abMltB showed the presence of 14 α-helices and four β-sheets [Fig. 1 ▸(*e*)]. It exhibited a typical domain composition with three distinct domains: an N-terminal domain (NTD), a catalytic domain (CD) and a C-terminal domain (CTD) [Fig. 1 ▸(*f*)]. The NTD consisted of two α-helices (H1 and H3), while the CD consisted of eight α-helices (H4–H11) [Fig. 1 ▸(*e*), (*f*)]. The CTD adopts the canonical MltB-domain structure containing a compact α/β fold in which a central curved four β-sheet is flanked by three α-helices [Fig. 1 ▸(*e*), (*f*)]. It is structurally interesting that the NTD is tethered to the CD by a long loop containing helix H3 that overlays the putative active site [Fig. 1 ▸(*e*), (*f*)]. Because the NTD is connected to the CD via a long loop that overlays the putative active site on the CD, a clear hole-like structure is observed in the surface model [Fig. 1 ▸(*g*)]. This structure forms a cavity measuring approximately 12.4 Å in width and 16.2 Å in height [Fig. 1 ▸(*g*)]. A cross-sectional view of this hole-like structure reveals that it extends to a depth of approximately 36.4 Å, forming a well defined tunnel-like cavity [Fig. 1 ▸(*h*)]. Analysis of the *B* factors reveals that most regions exhibit low *B*-factor values (around 32.5 Å^2^), indicating a highly compact structure. In contrast, the long loop connecting the NTD and the CD shows significantly higher *B*-factor values (around 89.7 Å^2^), suggesting that this loop is highly flexible and dynamically covers the putative active site [Fig. 1 ▸(*i*)].

Previous studies on SltB1, a soluble lytic transglycosylase that acts at a different position but is functionally related to MltB, have suggested that it may function as a dimer (Domínguez-Gil *et al.*, 2017 ▸). To gain a clearer understanding of the stoichiometry of abMltB in solution, we performed a size-exclusion chromatography coupled with multi-angle light scattering (SEC–MALS) analysis to determine its absolute molecular weight. The main peak corresponded to an experimental molecular mass of 40.1 kDa (1.8% fitting error), which indicated a monomeric composition of abMltB, considering that the theoretically calculated molecular weight of abMltB was 38.2 kDa [Fig. 1 ▸(*j*)].

### 3.2. Comparison of the structure of abMltB with its structural homologs

**Figure 2:**
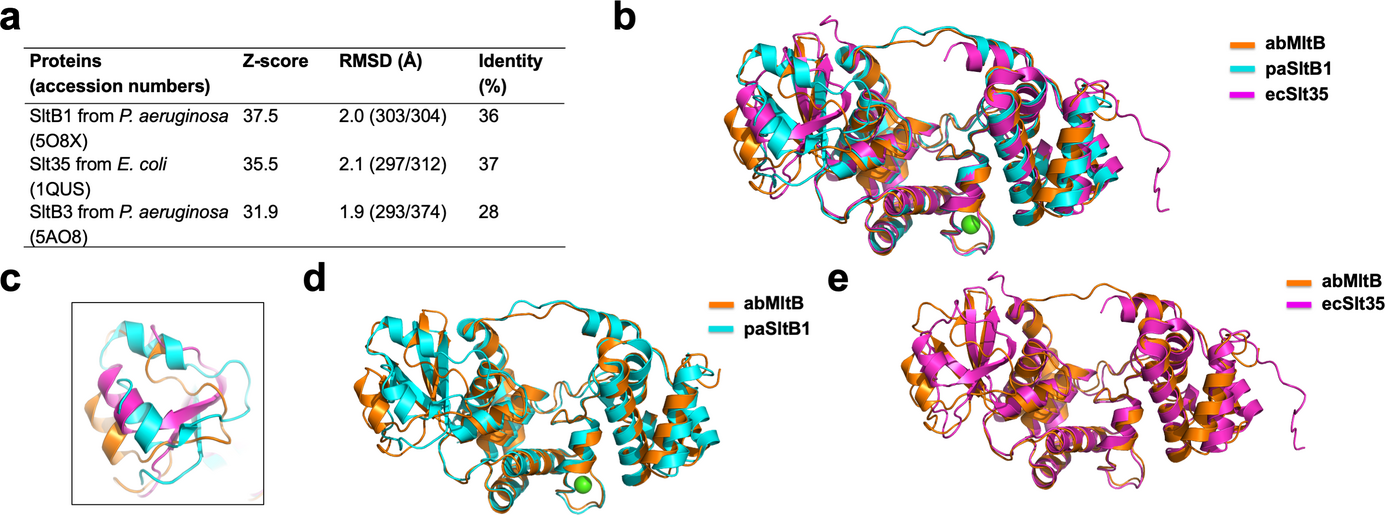
Structural comparison of abMltB with its structural homologs. (*a*) Results of DALI analysis. (*b*) Structure superposition of abMltB with its structural homologs, paSltB1 and ecSlt35. (*c*) Enlarged view of the CTD region, which shows the most prominent structural differences. (*d*) and (*e*) Pairwise structural superimposition of abMltB with paSltB1 (*d*) and ecSlt35 (*e*).

After analyzing the structure of abMltB, we compared it with MltB proteins from other species to identify structural similarities and determine which protein is most structurally similar to abMltB. To achieve this, we performed a structural similarity search using the DALI server (Holm & Sander, 1995 ▸). According to this analysis, SltB1 from *P. aeruginosa* (paSltB1), Slt35 from *E.coli* (ecSlt35) and SltB3 *from P. aeruginosa* (paSltB3) were identified as the top three structurally similar proteins [Fig. 2 ▸(*a*)]. These proteins are all soluble isoforms of MltB and belong to the soluble LT family, sharing approximately 28–37% amino-acid identity with abMltB.

The structural comparison performed by superposition of abMltB with biochemically well analyzed structural homologs (paSltB1 and ecSlt35) showed that the overall structure and domain composition were almost same as those of paSltB1 and ecSlt35, exhibiting an RMSD of 2.0 Å with paSltB1 and 2.1 Å with ecSlt35 [Fig. 2 ▸(*a*)]. Despite these structural similarities, pairwise comparisons revealed several clear structural differences. Notably, the CTD of abMltB did not perfectly overlap with those of SltB1 and Slt35 [Fig. 2 ▸(*c*)–(*e*)]. Compared with the CTDs of other homologs, the CTD of abMltB is positioned slightly farther away from the CD [Fig. 2 ▸(*e*)]. In the case of ecSlt35, the long loop connecting the NTD and the CD is missing from the model, likely due to its high flexibility, which made model building in this region unfeasible [Fig. 2 ▸(*d*)]. This observation supports our hypothesis that this loop region is highly flexible.

### 3.3. Putative active site of abMltB and its comparison with other lytic transglycosylases

**Figure 3:**
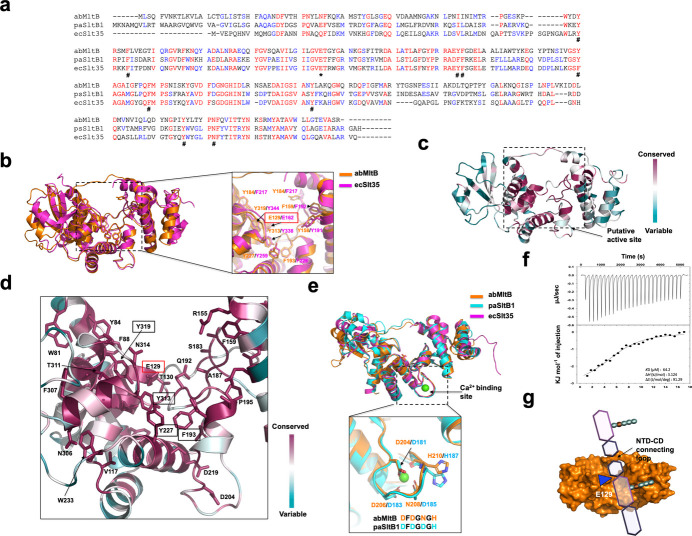
The tentative active site and working mode of MltB. (*a*) Sequence alignment of abMltB with its structural homologs, paSltB1 and ecSlt35. Mostly conserved and partially conserved residues are colored in red and blue, respectively. Hash marks indicate conserved residues that are involved in the substrate binding. An asterisk indicates the conserved glutamic acid at the catalytic domain of MltB, which is the critical residue for enzymatic reaction. (*b*) Active site comparison of ecSlt35 and abMltB. Eight residues that are involved in the substrate recognition are labeled. The most critical residue directly involved in the cleavage of the substrate, Glu162 (ecSlt35)/Glu129 (abMltB), is highlighted using a red box. (*c*) Cartoon representation of abMltB colored according to the degree of amino-acid sequence conservation analyzed by the Consurf server. (*d*) Close-up view of the tentative active site shown in Consurf analysis (*c*), highlighting the conserved residues. Residues predicted to be involved in substrate binding are marked with black boxes, while the residue expected to play a critical role in catalysis is indicated with red box. (*e*) Comparison of the Ca^2+^-binding site of abMltB with that of paSltB1. (*f*) The experimental result of the ITC experiment. (*g*) Tentative working model of abMltB. The location of NTD–CD connecting loop is indicated.

The structurally and functionally related enzymes ecSlt35 and paSltB1 have been relatively well studied (Domínguez-Gil *et al.*, 2017 ▸; van Asselt *et al.*, 1999 ▸). Therefore, we compared the sequence and structure of abMltB with those of these proteins by focusing on the active site to determine whether abMltB forms a similar active site. Sequence alignment revealed that Glu162, the key residue responsible for transglycosylation activity in ecSlt35, is conserved in abMltB as Glu129 [Fig. 3 ▸(*a*)]. Although the overall amino-acid identity is approximately 37%, several aromatic residues known to be involved in substrate sugar binding at the active site – such as Tyr117, Phe121, Tyr191, Phe192, Phe217, Phe226, Tyr259, Tyr338 and Tyr344 – are also completely conserved in abMltB [Fig. 3 ▸(*a*)]. These residues critical for enzymatic activity are also structurally located in corresponding positions [Fig. 3 ▸(*b*)]. Based on these observations, we speculate that abMltB likely carries out lytic transglycosylase activity through a mechanism very similar to that of ecSlt35. In addition to analyzing the composition of the active site, we further investigated the residues that are conserved across different species and potentially important for abMltB function by performing a residue conservation analysis using the Consurf server (Ben Chorin *et al.*, 2020 ▸). As expected, most of the residues conserved among MltB homologs from various species were concentrated around the putative active site [Fig. 3 ▸(*c*)]. Among the residues previously reported to be important for enzymatic activity or substrate binding in ecSlt35, Glu129 (catalytic residue), Tyr319, Tyr313, Tyr227 and Phe193 were identified as completely conserved in abMltB [Fig. 3 ▸(*d*)]. Additionally, several hydrophilic residues near the active site – such as Gln192, Ser183, Arg155 and Thr311 – were also found to be completely conserved [Fig. 3 ▸(*d*)]. These residues are likely to play important roles in the enzymatic function of abMltB.

Slt35, SltB1, and SltB3 are known to be Ca^2+^-binding proteins [Fig. 3 ▸(*e*)] (Domínguez-Gil *et al.*, 2017 ▸; van Asselt *et al.*, 1999 ▸; Lee *et al.*, 2016 ▸). The binding of Ca^2+^ has been reported to play a role in their proper folding and structural stability (Domínguez-Gil *et al.*, 2017 ▸; Kraft *et al.*, 1999 ▸; Lee *et al.*, 2018 ▸; van Asselt *et al.*, 1999 ▸). We examined the corresponding Ca^2+^-binding site in abMltB to determine whether any ion was bound, but no ion density was observed. Despite the apparent absence of Ca^2+^, abMltB appears to be properly folded and structurally stable, indicating that Ca^2+^ binding may not be critical for the folding or stability of abMltB. Although no bound ion was observed in the crystal structure, we examined whether the putative ion-binding site in abMltB is structurally similar to the Ca^2+^-binding site reported in other members of the LT family. To address this, we performed structural superimposition analysis. Remarkably, the Ca^2+^-binding motif identified in the paSltB1 study, characterized by the DxDxDxH sequence (Domínguez-Gil *et al.*, 2017 ▸), was found to be highly conserved in abMltB as a closely related DxDxNxH motif [Fig. 3 ▸(*e*)]. To further determine whether abMltB is capable of binding Ca^2+^, we conducted isothermal titration calorimetry (ITC) experiments. The results demonstrated that abMltB binds Ca^2+^ with a dissociation constant (*K*_d_) of 64.2 µ*M*, indicating moderate affinity [Fig. 3 ▸(*f*)]. Based on these findings, we concluded that abMltB indeed functions as a Ca^2+^-binding protein and that the identified motif may represent a *bona fide* Ca^2+^-binding site.

Based on these findings, we proposed a working model for the catalytic mechanism of abMltB [Fig. 3 ▸(*g*)]. The substrate is predicted to bind within a substrate-binding groove formed between the NTD–CD connecting loop and the CD, which is lined with multiple aromatic residues. The catalytic reaction is likely carried out via the conserved Glu129 residue through a transglycosylation mechanism. Although the precise function and significance of the tunnel formed by the NTD–CD connecting loop have not been clearly defined, it is plausible that this structural feature contributes to substrate positioning and selectivity.

## Supplementary Material

PDB reference: Membrane-bound lytic transglycosylase B from *Acinetobacter baumannii*, 9vlr

## Figures and Tables

**Table 1:** Data collection and refinement statistics ASU, asymmetric unit; RMSD, root-mean-square deviation.

Data collection	
Space group	*P*4_3_2_1_2
Unit-cell parameters	
*a*, *b*, *c* (Å)	75.49, 75.49, 175.36
α, β, γ (°)	90, 90, 90
Resolution range (Å)	29.31–1.79
Total No. of reflections	1 294 545
Unique reflections	48 657
Multiplicity†	26.6 (27.7)
Completeness (%)†	99.94 (99.96)
Mean *I*/σ(*I*)†	20.21 (1.88)
*R*_merge_ (%)†‡	12.13 (2.34)
Wilson *B* factor (Å^2^)	29.91
	
Refinement	
Resolution range (Å)	29.31–1.79
No. of reflections	48 651
*R*_work_ (%)	18.25
*R*_free_ (%)	20.18
No. of molecules in the ASU	1
No. of non-hydrogen atoms	2687
Macromolecules	2405
Solvent	282
Average *B*-factor values (Å^2^)	32.89
Macromolecules	31.60
Solvent	43.36
Ramachandran plot favored/allowed/outliers (%)	98.68/1.32/0
Rotamer outliers (%)	0
Clashscore	1.06
RMSD bonds (Å)/angles (°)	0.007/1.13

† Values for the outermost resolution shell in parentheses.

‡ *R*_merge_ = ∑_*h*_∑_*i*_|*I*(*h*)_*i*_ − 〈*I*(*h*)〉|/∑*_h_*∑*_i_I*(*h*)_*i*_, where *I*(*h*) is the observed intensity of reflection *h* and 〈*I*(*h*)〉 is the average intensity obtained from multiple measurements.
